# Pituitary Apoplexy Attributed to COVID-19 Infection in the Absence of an Underlying Macroadenoma or Other Identifiable Cause

**DOI:** 10.7759/cureus.13315

**Published:** 2021-02-12

**Authors:** Stephen J Bordes, Simone Phang-Lyn, Edinson Najera, Hamid Borghei-Razavi, Badih Adada

**Affiliations:** 1 Surgical Anatomy, Tulane University School of Medicine, New Orleans, USA; 2 Anesthesiology and Critical Care, Cleveland Clinic Florida, Weston, USA; 3 Neurosurgery, Cleveland Clinic Florida, Weston, USA; 4 Neurosurgery, Neurological Institute, Cleveland Clinic - Taussig Cancer Center, Cleveland, USA

**Keywords:** pituitary apoplexy, neurosurgery, covid-19, sars-cov-2

## Abstract

The novel coronavirus, severe acute respiratory syndrome coronavirus 2 (SARS-CoV-2), which causes coronavirus disease 2019 (COVID-19), is responsible for an array of extrapulmonary manifestations, including direct and indirect neurological complications. Currently, all published cases noting pituitary apoplexy in patients with COVID-19 have discovered underlying pituitary macroadenomas. Herein, we describe the first documented case, to our knowledge, of pituitary apoplexy attributed solely to COVID-19 in the absence of other identifiable causes. While much remains to be discovered and understood regarding COVID-19, we discuss the potential pathophysiology of COVID-19-associated pituitary apoplexy and raise awareness of this clinical complication.

## Introduction

Pituitary apoplexy is described as hemorrhage or infarction of the pituitary gland. It is a rare and sometimes fatal consequence of a pituitary macroadenoma. Pituitary adenomas are benign tumors of the pituitary gland that have an estimated prevalence of 6.2 cases per 100,000 individuals, and between 2-12% of patients experience apoplexy [1]. The sudden increase in pituitary gland volume secondary to ischemia or hemorrhage within the rigid sella turcica results in classic manifestations. These symptoms include sudden onset headache, cranial nerve palsies, and hypopituitarism [2]. However, pituitary apoplexy may occur in the absence of a macroadenoma as it can be attributed to other causes.

In 2019, the novel coronavirus, severe acute respiratory syndrome coronavirus 2 (SARS-CoV-2), which causes coronavirus disease 2019 (COVID-19), spread worldwide with symptoms ranging from an asymptomatic carrier state to acute respiratory distress syndrome and other extrapulmonary manifestations. The cause of such variation in symptomology remains widely unknown; however, it is important to be clinically aware of potential neurological complications.

Our case illustrates the clinical presentation of a patient with acute pituitary apoplexy one month after initial diagnosis with COVID-19 and highlights the importance of COVID-19 as a potential precipitating factor in the absence of other causes. Histopathological examinations of brain specimens of patients diagnosed with COVID-19 have shown acute hypoxic injury, with loss of neurons in the absence of thrombi or vasculitis [3]. Pituitary apoplexy has been attributed to COVID-19 within recent months; however, all of these cases have identified underlying etiologies such as pituitary macroadenomas [4,5]. To our knowledge, this case is the first in the literature to describe pituitary apoplexy, in the absence of a pituitary macroadenoma or other identifiable causes, as a sole complication of COVID-19 infection and its subsequent cerebral hypoxic injury.

## Case presentation

**Figure 1:**
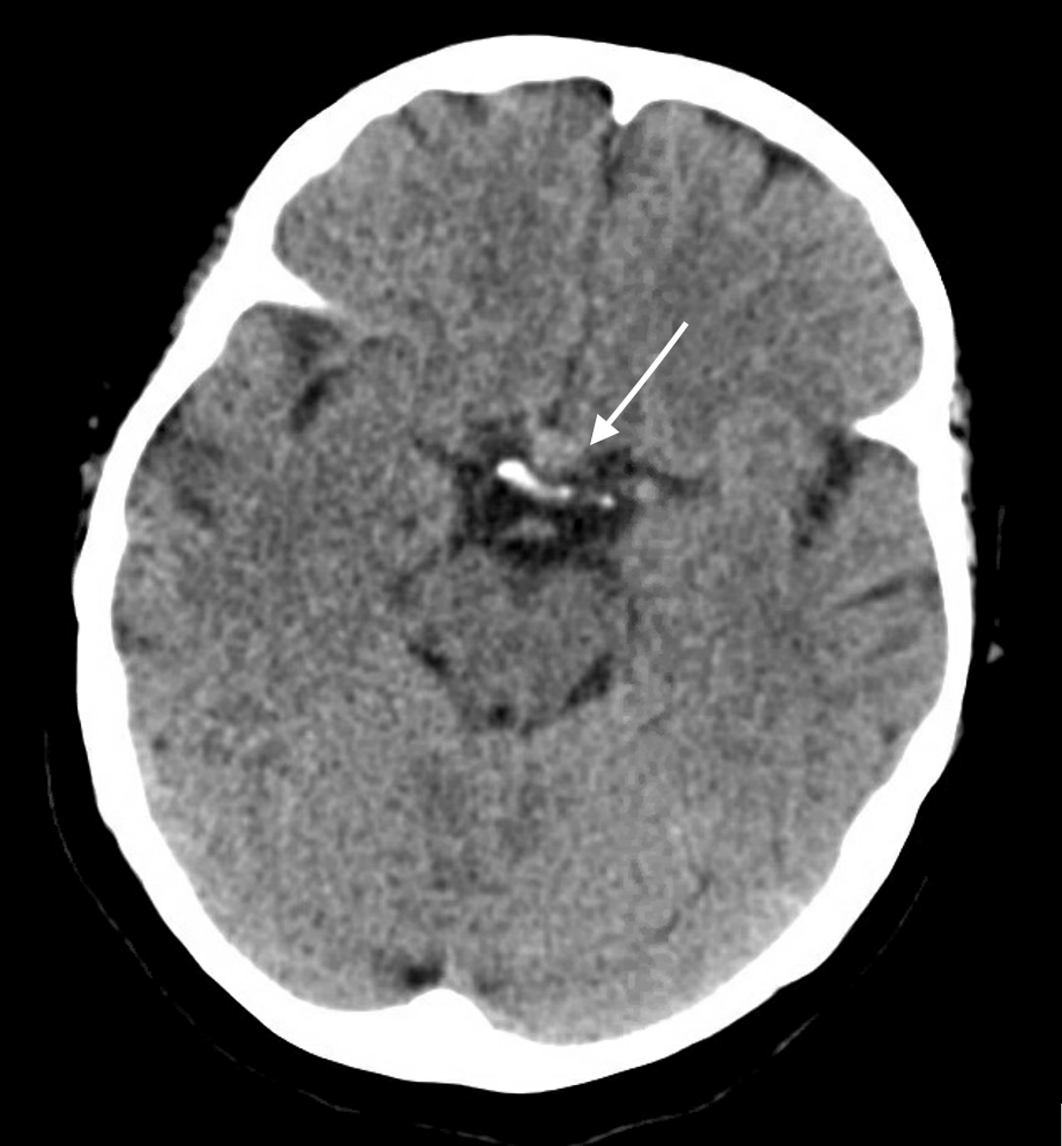
Axial unenhanced CT scan of the brain demonstrates an 8 mm sellar/suprasellar high-density lesion with mass effect on the optic chiasm (arrow) CT, computed tomography

**Figure 2:**
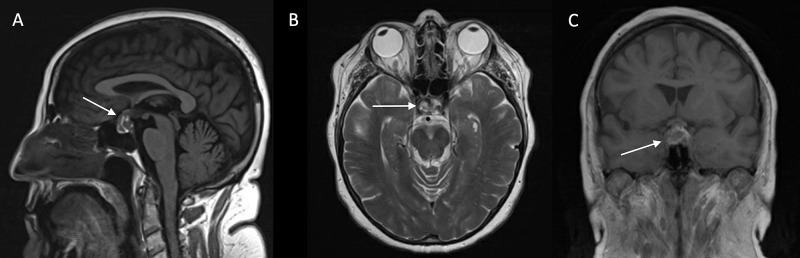
Initial MRI results Sagittal T1-weighted MRI (A) and axial T2-weighted MRI (B) images of the brain show high signal intensity suggestive of recent hemorrhage. Coronal gadolinium-enhanced T1-weighted MRI (C) revealing a 14 mm heterogeneously enhancing sellar lesion with suprasellar extension and effacing the optic chiasm. T1, longitudinal relaxation time; T2, transverse relaxation time; MRI, magnetic resonance imaging

A 65-year-old woman with a medical history of hypertension, fibromyalgia, and COVID-19 infection one-month prior came to the emergency department with complaints of a progressively worsening headache and persistent emesis for one week. The headache was described as frontal and retro-orbital. She also noted symptoms of nausea, vomiting, photophobia, and phonophobia. She endorsed continued malaise and cough associated with her previous COVID-19 diagnosis; however, her disease course was mild and limited to those symptoms. Initial neurological examination was unremarkable. She underwent a CT scan of the brain, which revealed an 8 mm sellar/suprasellar high-density lesion with possible mass effect on the optic chiasm (Figure 1). She was administered a single 10 mg dose of Decadron, and further imaging was ordered. MRI of the brain demonstrated a 14 mm heterogeneously enhancing sellar/suprasellar lesion with intrinsic high T1 signal areas and diffusion restriction, suggesting recent hemorrhage (Figure 2). An initial endocrinology workup was unremarkable for cortisol (1.6 ug/dL), thyroid-stimulating hormone (TSH: 3.28 uU/mL), and T4 (1.2 ng/dL). She had mildly elevated follicle-stimulating hormone (FSH: 30.8 mU/mL); however, luteinizing hormone (LH: 8.1 mU/mL) was not elevated as would be expected given her postmenopausal status. This finding was attributed to pituitary dysfunction. The patient had no evidence of diabetes insipidus. The endocrinology team started her on hydrocortisone three times daily. Over the course of her stay, the patient denied any other neurological symptoms and reported that her headache had significantly improved.

**Figure 3:**
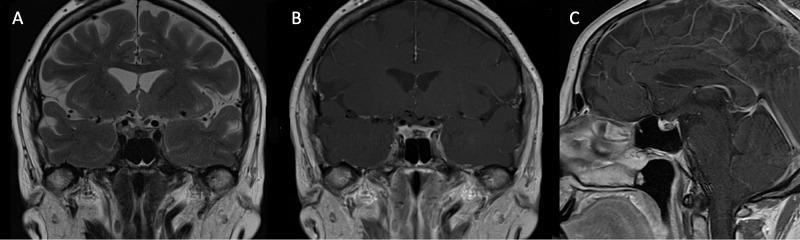
Follow-up MRI results Coronal T2-weighted MRI (A), coronal gadolinium-enhanced T1-weighted MRI (B), and sagittal gadolinium-enhanced T1-weighted MRI (C) demonstrate interval evolution of an intrapituitary hemorrhage, which drastically decreased in size, and show both subacute and chronic blood products with a new size of 8 mm x 4 mm. The pituitary gland is normal in size without extension to the suprasellar cistern or mass effect on the optic chiasm. T1, longitudinal relaxation time; T2, transverse relaxation time; MRI, magnetic resonance imaging

Approximately one month after discharge, the patient returned to the hospital for follow-up endocrinology laboratory measurements and imaging. She noted continued improvement of her symptoms and resolution of her headache. At this time, an MRI brain showed the interval evolution of an intrapituitary hemorrhage, which drastically decreased in size and showed both subacute and chronic blood products with a new size of 8 mm x 4 mm (Figure 3). Neurosurgery reviewed the images and noted the absence of a clear underlying pituitary tumor. Endocrinology reported normal prolactin levels (8.7 ng/mL) and adrenocorticotropin hormone (ACTH: 14.7 pg/mL). The patient had a slightly low LH (2.0 mU/mL) and FSH (6.0 mU/mL), both of which should be elevated due to menopause. The patient’s cortisol also remained low even while taking hydrocortisone. Thyroid hormones decreased while TSH was normal (T4: 3.3 ng/dL, Free T4: 0.4 ng/dL, TSH: 1.93 uU/mL). The patient was started on levothyroxine for central hypothyroidism. Endocrinology scheduled an additional follow-up for ACTH stimulation tests and urine studies due to hypernatremia (Na+: 149 mEq/L after fasting and water restriction). Neurosurgery scheduled a third MRI follow-up in six months (May 2021) to recheck for underlying neuropathology; however, at this time, the only precipitating factor and known association in this patient is a COVID-19 infection.

## Discussion

The novel coronavirus disease 2019 (COVID-19, caused by SARS-CoV-2) is known for its variety of clinical manifestations ranging from an asymptomatic carrier state to severe respiratory distress syndrome. While respiratory distress continues to be the most common manifestation of COVID-19, there are increasing numbers of reports describing extrapulmonary involvement, including neurological manifestations such as headache, dizziness, myalgia, anosmia, ageusia, stroke, encephalopathy, and Guillain-Barré [6]. Our patient is the first described, to our knowledge, to exhibit pituitary apoplexy following a COVID-19 diagnosis in the absence of other causes, including a pituitary macroadenoma. While the exact mechanism of such pathology remains unknown, we consider direct and indirect mechanisms herein.

The disease course of COVID-19 results in an initial insult on the lungs, triggering a severe inflammatory response. The stress of the viral infection on the body leads to increased sympathetic nervous system activation [7]. This activation can produce hematological and immunological changes in addition to an endocrine response. Abnormal stimulation of the pituitary gland can cause excess production of glucocorticoids. For example, a normal pituitary gland will enlarge 30%-45% during pregnancy, presumably due to increased estrogen causing lactotroph hyperplasia and an increase in pituitary blood flow. However, such enlargement does not clearly lead to an apoplectic event [8].

COVID-19 is recognized as a neuroinvasive virus and can cause an array of neuropathic manifestations such as headache, confusion, ataxia, dizziness, ischemic stroke, epilepsy, neuropathic pain, and myopathy [9,10]. The COVID-19 virus accesses host cells using an angiotensin-converting enzyme 2 (ACE2) receptor, which neurons and glial cells possess [9]. Furthermore, reports in the literature confirm the presence of COVID-19 virus in cerebrospinal fluid (CSF) in patients with severe COVID-19-associated encephalitis [10]. Patients with severe cases of COVID-19 are more likely to exhibit some of these concerning neurological symptoms, which have been attributed to immune-mediated mechanisms [9,10]. COVID-19 immune-mediated neuropathologies are further supported by positive patient responses to high doses of corticosteroids, as seen in our patient.

COVID-19 is further known to cause coagulopathies, the most common of which is characterized by elevated fibrinogen and D-dimer levels, mild thrombocytopenia, and a normal or mild prolongation of prothrombin time/activated partial thromboplastin time (PT/aPTT) [9,11]. COVID-19-associated coagulopathy has emerged as a hypercoagulable state more than a bleeding state; however, spontaneous hemorrhage is associated with thrombosis and remains a major complication. Pituitary apoplexy is associated with pregnancy, with some suggestion that there may be an association between a hypercoagulable state and gravid hormonal changes [12]. This can be extrapolated to an association between the hypercoagulable state seen in those diagnosed with COVID-19 and the occurrence of pituitary apoplexy in our patient.

## Conclusions

To our knowledge, this case is the first in the literature to describe pituitary apoplexy as a potential neurological manifestation of COVID-19 in a patient, one month after the initial diagnosis, in the absence of other identifiable causes such as a pituitary macroadenoma. While much remains to be understood regarding COVID-19, its mechanism of action, and its direct and indirect systemic effects, we aim to increase awareness of possible neurological complications in this patient population.
